# Urban Agriculture in Thailand: Adoption Factors and Communication Guidelines to Promote Long-Term Practice

**DOI:** 10.3390/ijerph20010001

**Published:** 2022-12-20

**Authors:** Sukanya Sereenonchai, Noppol Arunrat

**Affiliations:** Faculty of Environment and Resource Studies, Mahidol University, Salaya, Nakhon Pathom 73170, Thailand

**Keywords:** urban agriculture, Thailand, adoption factors, communication guidelines, long-term practice

## Abstract

The purpose of this study is to clarify influencing factors on the adoption and continuing practice of urban agriculture, and to propose communication guidelines to encourage more adoption and long-term practice. The psychological theories of the Theory of Reasoned Action (TRA), the Theory of Planned Behavior (TPB), and the Health Belief Model (HBM) were integrated to explain people’s behavior. Both quantitative and qualitative approaches were employed with 325 villagers of 13 communities in seven provinces of Thailand. The following techniques and instruments were used: a statistical analysis crosstab, stepwise multiple linear regression, one-way ANOVA, multinomial logistic regression, decision tree analysis, and descriptive content analysis using QDA lite miner software. The key results clearly show that attitude, perceived benefits, and perceived readiness were a significantly positive influence on those who adopted urban agriculture. Key drivers to villagers’ intention to continue practicing urban agriculture for the initiator group who own agricultural land were perceived to be behavioral control and social norm, while perceived readiness and communication played a crucial role for other groups to continue practicing urban agriculture. Communication guidelines to promote long-term urban agriculture practice can be designed based on the EAST framework, by making it easy, attractive, social, and timely, and through the Critical Participatory Action Research process.

## 1. Introduction

Urban agriculture is planting or causing enrichment in related processes and the distribution of food products, and it plays an important role in many aspects of urban development. This includes the use and reuse of resources, products, and services related to those activities that occur and exists in and around urban areas, or in the boundaries of the area that is being developed as a center to serve the people who live in the city [1].

Previous studies related to urban agriculture focused on the motives driving urban agriculture practitioners in: Canada and Ecuador using self-determination theory [2], Central and Eastern Europe [3]; Australia (focusing on demographics, gardening background, the physical elements of garden and garden inputs [4], Italy [5], and Malaysia, where undergraduates were included, as well as the Theory of Planned Behavior (TPB) [6,7].

To the best of our knowledge, the categorization of urban agriculture practitioners was only found in the study by Audate et al. [2], and this consisted of four groups: the eco-engaged (young adults mostly with university degrees and stable employment), the socio-engaged (who are seeking collective benefits), the econo-experts (who have prior experience in agriculture and completed high school), and the versatile caretakers (women with children who are head of the household). This categorization aimed to analyze the interrelation between intrinsic and extrinsic motivations, relying on personal and socio-economic factors. 

Urbanization and land use changes are considerably influenced human activities and environment condition [8]. According to urbanization in Thailand 2021 [9]. Thailand’s total population lived in urban areas and cities around 52.16 % in year 2021, which increased from year 2011 (41.7%). However, Thailand’s urbanization rates are still low compared to other developed nations, which reached 80–90%.

The concept and practice of the ‘urban vegetable garden’ or ‘urban agriculture’ in Thailand was promoted since 2010 by the Sustainable Agriculture Foundation (Thailand) in collaboration with the Foundation for the Media Center for Development, the Urban Agriculture Training Center, and the Urban Vegetable Garden Network with the support of Thai Health Promotion Foundation. Based on a shared awareness of the problems in terms of food security, health, society, and the environment of urban people, the organizations’ collaborative aim is to enhance the food self-sufficiency of urban people [10]. 

In 2019, the importance of solving food insecurity in the city became a critical one because for the first time, Thailand had a larger urban population than those living in rural areas, thus reflecting the expansion of the city. The urban population is responsible for less than 10% of their own food production, and thus it became important for the Ministry of Agriculture and Cooperatives to accelerate the development of urban agriculture, especially during the COVID-19 pandemic, and also in terms of the next era that must focus on the urban ecosystem with regard to people’s health and city quality [11].

Therefore, this study aims to analyze factors influencing people’s adoption of urban agriculture, their intention to continue practicing, and communication guidelines to encourage people’s adoption and continuing urban agricultural practice. To fill the gap of previous research, typologies of urban agriculture were also highlighted in order to understand insight and perception of each group. Moreover, an integrated framework was employed in this study, which was developed from three theories: the Theory of Reasoned Action (TRA) and the Theory of Planned Behavior (TPB) as behavioral predictor theories [12,13]; the framework also included the Health Belief Model (HBM) [14], as the benefits of urban agriculture also relate to human health. This study can contribute to better understanding of factors driving different groups of people to adopt urban agriculture and communication guidelines for promoting urban agricultural practice. The following details were elaborated into four sections consisting of: literature review, materials and methods, results and discussion, and conclusions and recommendations. 

## 2. Literature Review

### 2.1. Integrating the TRA, TPB, and HBM to Understand Villagers’ Decision

The TRA suggests that a person’s attitude toward behavior and subjective norms can lead to their intention and their performance of a behavior [15]. The TPB was developed [13] to better understand a person’s behavior, including perceived behavioral control (PBC) as another factor apart from TRA. PBC reflects the state of people believing in their ability to perform a behavior. TPB was used to understand volunteering in urban agriculture [7]. In the urban agriculture context, this theory implies that a combination of an individual’s attitudes toward volunteering for urban agriculture, perceived social pressure by others, and perceived control beliefs can predict the individual’ s intention to volunteer to participate in urban agriculture. 

**Figure 1:**
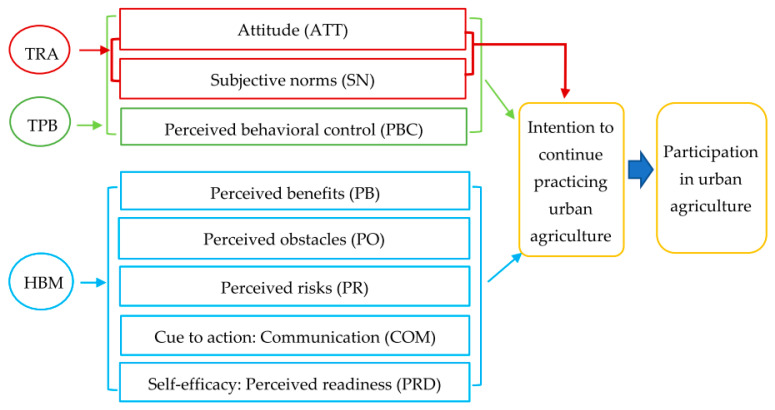
The integrated psychological model employed in this study.

Urban agriculture also contributes to better health conditions, as mentioned in previous studies [16,17,18,19,20]. Therefore, the Health Belief Model (HBM), developed by social scientists at the US Public Health Service in the early 1950s [14], was also adopted in this study to develop the influencing factors analysis. The HBM suggests that humans’ belief in the threat to their health and the effectiveness of the suggested health behavior will predict the tendency of them to adopt the behavior. The main elements of HBM consist of: (1) perceived susceptibility (perception of the risk); (2) perceived severity (perception of the seriousness); (3) perceived benefits (perception of the effectiveness of various actions); (4) perceived barriers (perception of the obstacles to performing a recommended action); (5) cue to action (stimulus needed to adopt a recommended action; and (6) self-efficacy (person’s confidence to successfully perform a behavior). The integrated model employed in this study is demonstrated in Figure 1.

### 2.2. Factors Influencing People’s Participation in Urban Agriculture

In Ecuador, a qualitative method for data collection and analysis was employed, and the study found that self-provision of healthy food, health and wellbeing, empowerment, social capital, and economic rewards were discovered to be the urban population’s motivation for participation in urban agriculture participation [21]. Similarly, Pourias et al. [22] emphasized that not only the provision of, and access to, fresh food through urban agriculture can be significant for gardeners in high-income countries, but that personal well-being and social capital also appear to be more significantly involved [23]. Using hierarchical regression to understand Malaysian youth volunteers participating in an urban agriculture program, Tiraieyari and Krauss [7] employed Structural Equation Modelling (SEM), and observed attitudes toward urban agriculture, subjective norms, career motives, and perceived barriers to participation, respectively. In Australia, home gardeners highlighted food production, enjoyment, and health as their leading motivators, while enjoyment and connection to others were found to be more important for community gardeners [4]. Ruggeri et al. [5] proposed that personal well-being, pleasure, and the desire for more nutritious fresh food were the key motivators for citizens of Milan. For home gardeners in Toronto, apart from self-satisfaction, access to fresh food, education, environmental awareness, and aesthetics also played a crucial role [24]. 

## 3. Materials and Methods

### 3.1. Study Areas

**Figure 2:**
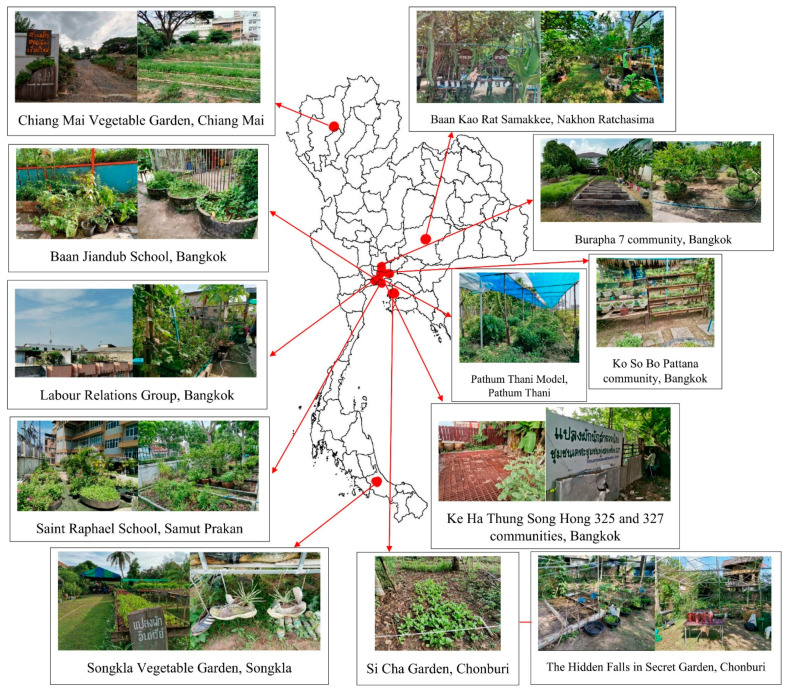
Study areas.

The study areas were purposively selected covering 13 communities in seven provinces of Thailand consisting of Bangkok, Samut Prakan, Pathum Thani, Chonburi, Songkla, Nakhon Ratchasima, and Chiang Mai (Figure 2). Based on recommendations from key persons involved with the UVGP, who initiated and transferred knowledge about urban agriculture, the list of nine communities were recommended under their UVGP because of their continuous practice, and their attempts to deal with difficulties and challenges during their urban agriculture projects. Although a few communities could not implement community farming due to the inconvenience of the area, some of the villagers tried to continue urban agriculture in their homes. Meanwhile, another three communities not joining UVGP were also recommended as the communities’ strengthening in urban agriculture and were broadcasted on the news. 

Overall, the study areas were urban communities, where the main areas were occupied by residential and business spaces. The major social status and lifestyle of people in the urban communities were that of enjoying the practice of agriculture to spend time more beneficial and the agricultural productivity can be their own food and share to other households. The trend of practicing urban agriculture in the study areas became more popular especially due to the spreading of COVID-19, causing most of them to have more time at home, and lack of food for some time.

### 3.2. Sampling Design and Data Collection 

Purposive sampling was carried out of the key individuals who initiated and/or were opinion leaders in the practice of urban agriculture inside their communities, and they were interviewed. Furthermore, a snowballing technique was employed with the key individuals and opinion to link with other villagers who participated in urban agriculture. There was also accidental sampling of villagers in each community who were available and willing to provide their information; they were interviewed following a questionnaire. Thus, with 25 villagers from each community, a total of 325 responses were collected, analyzed, and synthesized to achieve the study objectives.

The questionnaire was designed based on three theories consisting of TRA, TPB, and HBM. The reliability of the questions was tested with regard to psychological factors. Cronbach’s alpha was between 0.845 and 0.964. Consequently, the questions were reliable and fit for the objectives of this study (Table 1).

The questionnaire was validated by three experts in the fields of environmental studies and statistics, agricultural communication, and environmental psychology. Subsequently, it was pre-tested with 10 villagers in the study areas, although they were not asked to take part in the final study. A few questions and some of the language used in the questionnaire were revised to make them easier to understand based on the suggestions of pilot villagers.

The questionnaire consisted of three main parts (Table 2): (1) demographic information, (2) social and physical distance factors, and (3) psychological factors. A checklist, an open form, and a five-point Likert scale were employed to record their responses, where 5 = maximum and 1 = minimum scores.

### 3.3. Data Analysis

To decode villagers’ motivations to participate in urban agriculture, data analysis using SPSS Statistics Software version 22.0 was used consisting of: the normal distribution test, test of homogeneity of variances, multinomial logistic regression, one-way ANOVA (using post hoc multiple comparisons, Dunnett T3), stepwise multiple linear regression, crosstab, Pearson’s correlation and multinomial logistic regression. Nine psychological factors covering attitude (ATT), social norm (SN), perceived behavioral control (PBC), perceived benefits (PB), perceived risks (PR), perceived obstacles (PO), perceived readiness (PRD), communication (COM), and intention (ITT) to practice urban agriculture were highlighted in this study. 

The qualitative data from the villagers’ interviews were analyzed by thematic content analysis [25]. The recorded data were transcribed, reviewed, and classified following the study objectives. After that, the QDA Miner Lite Program was used to code, label similar meanings, and to group the data [26]. The overall results were reviewed by each researcher, and then with face-to-face discussions between two researchers. 

## 4. Results and Discussion

### 4.1. Demographic Information

Most of the respondents were female (around 61%), and did not own farmland (74%). Their average age, schooling, and numbers of year practicing urban agriculture were: 49, secondary school, and 3 years, respectively. Five groups of the villages participating in urban agriculture were found in the study area consisting of: (1) farmland owner–initiator–farmer–beneficiary (8.6%), who owned their agricultural area including initiated urban farming in their community and gained benefits from their farming; (2) initiator–farmer–beneficiary (16.3%), who initiated urban farming in their community and gained benefits from their farming, but did not own agricultural area; (3) participator–continuator–beneficiary (16.9%), who participated in urban agriculture and continue to do urban agriculture at their home including gained benefits from their farming; (4) participator–beneficiary (10.5%), who participated in urban agriculture and gained benefits from their farming; and (5) beneficiary (47.7%), who gained benefits from urban agriculture, but did not own and did not participate in urban agriculture. 

### 4.2. Villagers’ Participation in Urban Agriculture and Their Perceptions 

To understand the motivation of five groups of the respondents for participating in urban agriculture, multinomial logistic regression (MNL), one-way ANOVA, crosstab, and Pearson’s correlation were analyzed. A multinomial logistic regression model was analyzed to explore factors influencing people’s decisions to participate in urban agriculture. 

First, to confirm that no multicollinearity was found with all predictors, a linear model was tested to observe the values of Tolerance (TOL) of more than 0.1, and a variance inflation factor (VIF) of less than 10 between independent variables [27]. The mean of TOL and VIF were 0.618 and 1.963, respectively. 

The model fitting information showed a good model fit with the chi-square ratio test of 731.861 (*p* = 0.000). The pseudo R-squared values were as follows: Cox and Snell: 0.895, and Nagelkerke: 0.95), meaning that the independent variables in this study were an influence on villagers’ participation in urban agriculture with 95.3%. These values were also accepted because the higher pseudo R-squared values model reflected a better prediction of the outcome. The accuracy of the multinomial regression model was correctly classified as 90.2% (Table 3), reflecting the appropriate model and being able to project future estimates.

These are the likelihood ratio tests (Table 4) for the effects of the model and the partials whose low *p*-values show the high significance of the variables in the model.

The marginal effect results of factors influencing people’s decision to participate in urban agriculture, when the reference category was group 5, were presented in Table 5. There are more details of each factor below.

(1) Attitude (ATT)

The MNL analysis with regard to the ATT of group 1, 2, 3, and 4 was found to be a significant positive influence, where the highest value was group 1 (Exp(B) = 6,368,265.704 ***), followed by group 3 (Exp(B) = 2,396,974.203 **), group 4 (Exp(B) = 1,077,220.039 **), and group 2 (Exp(B) = 853,810.774 **), and βs for all these groups were found to be positive. This means that attitude was the crucial factor influencing people’s decision to practice urban agriculture, particularly those who were the initiators. The result of one-way ANOVA analysis to explore differences of attitude toward urban agriculture was also found to be in a similar direction. The ATT of group 5 respondents were found to have significantly negative differences compared with other groups. These results are in strong congruence with Tiraieyari and Krauss [7], who stated that attitude was the strongest factor influencing students’ participation in urban agriculture.

Based on crosstab and mean analysis to generate clear detail of each sub-factor of ATT, it was strongly demonstrated by the results that the group 5 respondents, compared with other groups, perceived the least score, particularly the relationships among WEFP resources (ATT5, average score of 2.51). This could be due to their low connection with practical urban agriculture; conversely, more connection could potentially promote higher perceived benefits, as also suggested by Pourias et al. [22], Rogge and Theesfeld [28], and Soga et al. [23]. Thus, social connectedness could promote more interaction among the community.

Group 5 respondents connected with urban agriculture mainly by gained products or food from other groups’ cultivation, obviously during the spread of COVID-19. This may be connected to their highest average score for the perception of the strengthening of social relationships in their community (ATT4, average score of 3.34). Many of them also gained knowledge and information about urban agriculture, but did not pay much attention to it, as they had no need to employ it at the time. The social benefit was also in line with the findings of Kingsley [16]; Pourias et al. [22]; Rogge and Theesfeld [28]; and Soga et al. [23], in that the practice of urban agriculture could promote more interaction among the community members.

The example of PB sharing from group 2 respondents in Chiang Mai province shows urban agriculture practice as not only being a food source (ATT3), but also a public space and a learning center for urban residents (ATT4). It is an agricultural education area for students and adults from both inside and outside the province, a green public space which is easily accessible to the residents of Chiang Mai, and which includes participation connecting people from multi-sector networks. The networks connection was established when the site was changed from a waste dump to an agricultural area, where the population began learning how to grow vegetables by adjusting the soil properties to fit with their plants. 

The average perception score of the relationships among WEFP resources (ATT5) was clearly observed to be the lowest average score and the lowest score within almost all groups of respondents, except for group 1. Therefore, the question of how to promote better understanding of this point (the relationships among WEFP resources) is still an opportunity for further work.

(2) Perceived behavioral control (PBC)

Compared with group 5, perceived behavioral control (PBC) was found to be a significantly negative influence on groups 1, 2, 3, and 4 (Exp(B) = 2.962 × 10^−7^ **, 8.528 × 10^−8^ **, 7.339 × 10^−9^ **, and 2.604 × 10^−6^ *, respectively). This might be due to their occupations, as groups 2 to 5 were found to be mainly employees, and the percentage of respondents who were employees was 36.69%. They explained that their time to learn and practice urban agriculture also needed to be based on the agreement of their employer. If there were more tasks to finish, they would have to attend to the first priority in their job. 

The results of crosstab analysis also highlight the point that group 5 perceived the least (score = 2) for all sub-elements of PBC (% of total for PBC1, PBC2, and PBC3 were 31.4%, 36.3%, and 36.3%, respectively). Moreover, the results of one-way ANOVA of the PBC for group 5 compared with other groups were also found to be a significantly negative influence. This could reflect the point that the urban agriculture initiators and adopters perceived their self-control, and felt more confident in continuing with urban agriculture than those who only gained benefits from urban agriculture without practicing it.

The average of PBC1, PBC2, and PBC3 showed that the results of the group 5 respondents are the lowest, especially for personal control in urban agriculture (PBC2, 2.24) and their own decision whether to practice urban agriculture or not (PBC3, 2.24). In contrast, the results of group 1 respondents are the highest scores, in particular for their confidence in continuing urban agriculture (PBC1, 3.50), and their average score of PBC was the highest (3.18) compared with other groups. This seems to be an opportunity to promote their confidence, by supporting practical knowledge training and relevant materials in order to drive them to be an agent of change, and to introduce and persuade other groups to participate in practicing urban agriculture.

(3) Perceived benefits (PB)

In comparing PBs of the other groups with group 5 respondents, it is clear that PB played a crucial role for group 1 to 4 respondents to participate in urban agriculture (Exp(B): 55,628,264.015 **, 352,811,535.239 **, 1,352,429.342 *, and 233,157.646 *, respectively). The analysis of one-way ANOVA clearly showed that the perceived benefit of food waste reduction (PB4) by group 1 to 4 respondents were found to have significantly positive differences (1.19908 *, 1.02118 *, 0.94194 *, and 0.81252 *, respectively) compared with group 5. The respondents of groups 1, 2, 3, and 4 all agreed that they could collect the vegetables from their urban agriculture area in the amounts they wanted for cooking based on the menus and numbers of their family members. If they bought the vegetables from a market or a superstore, they were also forced to buy the packaging of that shop. Sometimes, they only needed a few vegetables, but they could not buy such an amount, so there were some vegetables left in the bin. Food waste is also related to the environmental aspect, in complete accordance with Sroka et al. [19], who highlighted that improving the environment was an influential stimulation to adopt urban agriculture. In terms of PB, eating safe food (PB1) was additionally found to have the highest average score among all groups (3.84). The benefit of finding safe food consumption supports the results of Opitz et al. [29] and Gray et al. [30].

In contrast, less benefits were perceived by group 5 with the lowest average score for all sub-elements compared with other groups, particularly income generation (PB6, average score: 1.10) and value-added creation (PB10, average score: 1.23). This is in accordance with Kirby et al. ’s findings [20], that the type of urban agriculture participants was influenced by perceived impacts and motivations. Those who were the owners or initiators of urban agriculture demonstrated greater wellbeing than employees.

With regard to the details of each element of PB, group 2 respondents were found to have the highest average score for having eaten safe food (PB1, average score: 4.43), promoting more exercise (PB2, average score: 4.13), and having the opportunity to learn more (PB7, average score: 4.09). Group 2 respondents who worked as employees in Bangkok emphasized the benefit of having eaten safe food, particularly during the serious COVID-19 pandemic, which led to temporary unemployment causing shortages of money and food. There was sufficient food, which was also safe, from their own urban agriculture practice (PB1). They also gained relevant knowledge in planting and caring for a vegetable garden, which will be a source of sustenance for their whole lives (PB7). Another important benefit was the contribution to their mental health (PB5), as they were able to relax after work. Respondents from one community explained that collecting snails and worms from the vegetables relaxed them and they realized how plants grow. In addition, they complimented each other for growing the vegetables so well.

Moreover, the benefit of spending free time more constructively (PB3) and creating value added (PB9) to groups 1 to 4 compared with group 5 respondents were also shown to have significantly positive differences. The respondents from Chonburi province clearly highlighted these benefits, particularly during the serious spread of COVID-19, which caused a cessation of activity and no space for their children to play, due to the fact that some public areas were closed. Urban agriculture is a space within which their children can play and learn from their surrounding environment (PB3, PB7). Moreover, the respondents who were housekeepers or unemployed, and made baked goods for sale, could use their vegetables and certain types of flowers to add value to their bakery (PB9). They could also earn more income from the vegetable and flower bakery (PB6). This also motivated their children to enjoy eating vegetables, as it was safe food (PB1) that they themselves grew (PB8). Furthermore, the Bangkok respondents focused on the point that urban agriculture helped them to relieve stress during temporary unemployment and the stress caused by the pandemic (PB5), which is also in line with the findings of Hofmann et al. [17]. They also engaged in more exercise and thus improved their physical health (PB2). Some respondents indicated the differences before and after engaging in urban agriculture, stating that after they now had better fitness and a firm physique, in strong accordance with the findings of Zick et al. [31]. 

Conversely, the benefit of the value-added creation of all groups was found to be the least average scores (PB10, average score from 5 groups = 1.72) among those ten benefits, followed by income generation (PB6, average score from 5 groups = 1.84). The result of less perceived benefit in the terms of economic or income generation (PB6) was also consistent with the finding of Kirby et al. [20]. The strengthening of relationships in a community (PB9) or socialization, especially for group 1 respondents is also similar to the findings of Pourias et al. [22], Rogge and Theesfeld [28], and Soga et al. [23].

This is an opportunity to promote more values of urban agriculture through communication. Learning from some communities, as their reflection of value-added products was also a practical idea, they used certain kinds of vegetables and edible plants to cook and bake in order to sell such items as sandwiches, burgers, and coconut rice pancakes. Ideas such as this could inspire other kinds of cooking, based on the social capital within each community, which could also generate more income and promote the identity of that community.

(4) Perceived Readiness (PRD)

The result of MNL analysis, when the reference category was group 5, PRD of group 1 was obviously indicating the most significant factor influencing their decision to begin urban agriculture (Exp(B) = 638,201,298.217 ***), followed by group 2 (Exp(B) = 68,255,949.265 **), group 3 (Exp(B) = 1,076,712.577 **), and group 4 (Exp(B) = 173,003.759 *), respectively. The respondents perceived their readiness or self-efficacy, especially due to gaining support from the Thailand Sustainable Agriculture Foundation (SAF), in aspects of knowledge and materials to produce bio-fertilizer, equipment for farming in the city, and seeds. They stated that the vegetable plot needed to be planted because of factors to do with light and duration of planting. If they needed advice, they could contact the staff at SAF for further guidance.

One-way ANOVA analysis of group 5 respondents for PRD had significantly negative differences compared with other groups, while group 1 respondents seemed to be the readiest for urban agriculture, as the highest scores of all resources were mainly found from group 1, particularly with regard to farming area (4.79) and knowledge (4.61). Analyzing each sub-component in detail within PRD using crosstab also highlighted similar results to the effect that most of group 5 respondents rated all sub-elements of PRD as the lowest score compared with other groups. 

Regarding the average score of PRD, having knowledge (PRD8) was perceived as the highest score for almost all groups; the lowest score (1.59) was found from group 5. Accordingly, to promote more adoption of urban agriculture, the existing knowledge could be the opportunity to link the respondents’ current knowledge to connect with how to really practice, which is also in strong agreement with the findings of agricultural communication [32]. 

However, alternative energy, such as solar energy, should be supported in urban agriculture, particularly for those who need to pump water as a resource for their planting. The respondents from all groups rated energy readiness as the lowest score (the total average score was 1.47) compared to other resources for urban agriculture.

(5) Perceived risks (PR)

The factor of PR, compared with group 5 respondents, was found to have a significant negative influence on group 1, 2, 3, and 4 respondents (Exp(B) = 5.314 × 10^−9^ *, 4.566 × 10^−9^ *, 7.699 × 10^−11^ **, and 7.554 × 10^−10^ *, respectively), implying that perceived higher risks of urban agriculture could reduce the tendency for people to adopt urban agriculture, especially those who did not participate in urban agriculture. 

This was also similar to the result of one-way ANOVA analysis of PR for group 5 compared with groups 3 and 4, showing significantly positive differences (0.55298 * and 0.40557 *, respectively). Similar to the results of Sroka et al. [19], it was found that those who were more relevant (with a low social distance) and were less affected by hazards to urban agriculture would be more dedicated to this practice. Moreover, the crosstab analysis of PR clearly reflected less risk perception for all groups of the respondents; mainly the highest scores of each sub-element for all groups were rated as strongly disagree (score = 1) and disagree (score = 2). 

Group 5 respondents did not really perceive a high risk of urban agriculture, their perception scores for all sub-elements under PR were disagreement (score = 2), and their average PR score was only 1.97. They supported this lesser perception, as realized by others’ practices inside their community. They hardly noticed the traffic jams from agricultural machines and waste on the road, insects that harmed humans, the deterioration of the surrounding environment, the threat to human health, the spoiling of the urban landscape, and economic development slowing down. In addition, group 1 to 4 respondents emphasized that they tried to employ bio-fermented substances and avoid chemical fertilizers, which would not affect the surrounding environment and human health, both directly from contamination of the food and the environment. Furthermore, their agricultural areas were mainly set as a separate zone for agricultural practice, where the traffic was not impeded and the scenery of the urban landscape was not impacted in their community. As Thailand’s economy is mainly based on the agricultural sector, most of the respondents perceived less risk of a static economy from urban agriculture. They realized the benefits of urban agriculture not only in terms of food security for their households, but also how it could be sold to other households and countries.

(6) Perceived Obstacles (PO)

The MNL analysis of PO was mainly found to have a significantly positive influence on group 4 respondents referencing with group 5 (Exp(B) = 44.602 *). Similarly, the results of the one-way ANOVA of group 5 also found significantly positive differences with group 1 to 3, except for group 4 respondents. It can be implied that higher PO could reduce the tendency of practicing urban agriculture. The highest average PO was found from group 5 respondents (3.34), especially not having enough time for urban agriculture (PO1, 3.43). This might be due to the majority of them working as an employee in a company or a factory, as they mainly spend their time working. The highest score of sub-elements within PO was found from group 4 respondents mentioning their family responsibility (PO3, 3.47) because they need to take care of their family members in addition to their work as well. 

From the average PO score from all groups, comparing among all obstacles, lacking of knowledge (PO4) showed the highest average score (2.97). At the same time, communication factor was not found as a key factor influencing all respondent groups to practice urban agriculture. Accordingly, promoting attractive, appropriate, and accurate messages can be an opportunity for villagers to adopt and continue practicing urban agriculture.

(7) Decision tree analysis of demographic and social and physical distance factors

Decision tree analysis of data from 325 respondents, with participation as a dependent variable, and independent variables of sex, age, education, occupation, accommodation characteristics, living, distance, and agricultural area were proved. The variables to predict influencing factors on participation characteristics of practicing urban agriculture showed 55.7 percent correctly (Table 6), while the risk of this prediction showed 44.3 percent (Table 7).

**Figure 3:**
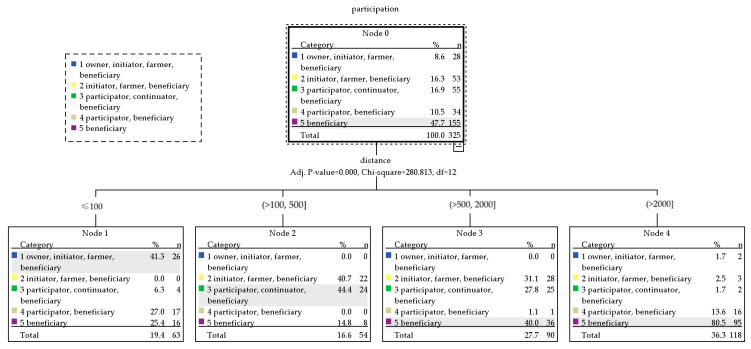
Decision tree analysis.

Distance seemed to be an obvious factor influencing groups 1, 3, and 5. The results express rules for three classifications as follows (Figure 3). The nearest distance between group 1 respondents’ household (less than 100 m) and their agricultural area presented is a key factor influencing their practice of urban agriculture (41.3%), group 3 (44.4%), while the farthest distance of more than 2000 m could cause no participation for group 5 respondents (80.5%).

### 4.3. Factors Influencing Villagers’ Intention (ITT) to Continue Practicing Urban Agriculture

Stepwise multiple linear regression was employed to analyze factors influencing the ITT of each group of respondents (Table 8) to practice urban agriculture. 

PBC and SN positively influenced group 1 respondents to initiate and continue practicing urban agriculture. This was supported by the respondents’ explanation that they previously and continuously practiced agriculture on their own land. Then, particularly during the COVID-19 pandemic, they talked to their friends and neighbors, and found that others were facing food insecurity, such as food shortages, and that it was difficult to deliver and go out to buy. At the same time, after seeing this information, the project launched by the Sustainable Agriculture Foundation, they consulted their close associates about submitting the community urban agriculture project. These prior conditions of previous practice and food insecurity problems were key starting points for people’s interest in urban agriculture. It was also mentioned that people’s desire for food led to them undertaking urban agriculture [5]. Their ability to produce food enhanced their levels of self-efficacy [33]. 

Based on correlation (r) analysis, as owners of the farming area, their PRD in all aspects were found to be positively correlated with PBC consisting of: human resources (r = 0.617 **), materials and equipment (r = 0.591 **), seed (0.580 **), water resource (0.555 **), bio-fertilizer (r = 0.554 **), area (r = 0.501 **), knowledge (r = 0.403 **), and alternative energy (r = 0.266 **). These correlation results reflect the fact that their higher readiness for urban agriculture in all aspects made them perceive more self-efficacy in their practice of urban agriculture. Consequently, the central and local authorities needed to launch supporting mechanisms to drive or promote readiness to the people. 

SN played a crucial role regarding the intention of both group 1 and 2 respondents to continue with urban agriculture. Therefore, SN should be promoted to enhance the greater intention of the people, which is similar to Mayne et al. [34], who suggested methods to raise SN by: (1) using persuasive advocates, such as role models and opinion leaders, who could influence people’s decisions; (2) providing social proof that was relevant to others; (3) providing information comparing their behaviors with their neighbors to ensure their normative, desirable, and undesirable behaviors; and (4) spreading urban agricultural practice as a new social norm by linking the present or creating new reference groups.

Group 2 respondents realized their readiness and ATT, although they perceived some obstacles, based on SN driving their decision while gaining information and knowledge from COM. The results clearly prove that five factors were found to be a positively significant influence on group 2 adoption of urban agriculture consisting of: perceived readiness or PRD (t = 3.310, *p* = 0.002), ATT (t = 2.587, *p* = 0.013), SN (t = 3.218, *p* = 0.002), and COM (t = 2.170, *p* = 0.035). Meanwhile, PO (t = −6.665, *p* = 0.000) was found to negatively influence their decision.

COM also played a crucial role in motivating the respondents of group 2 (t = 2.170, *p* = 0.035), group 3 (t = 2.225, *p* = 0.030), and group 4 (t = 3.609, *p* = 0.001) to adopt urban agriculture. As explained by some respondents from group 2, 3, and 4, they got to know and learned practicing urban agriculture from group 1 respondents, agricultural experts, or experienced farmers both online and onsite training and study visit. To strengthen the confirmation of the practice of urban agriculture by the villagers, communication to promote perception of its relative advantages, the compatibility of it with their current practice and living, less complexity, trialability, and observability should be emphasized [35]. Moreover, social media should be utilized as an effective channel of communication, especially during the serious spread of COVID-19, to open more accessibility of the practical knowledge at their available time and place.

The results clearly show that the PRD of group 2 (t = 3.310, *p* = 0.002), group 3 (t = 5.906, *p* = 0.000), and group 4 (t = 2.907, *p* = 0.007) were positively significant in terms of ITT. PRD, or self-efficacy, was also important because this factor can ensure people’s confidence in practicing urban agriculture. In environmental contexts, self-efficacy consists of perceived self-capability and abilities to behave. In order to promote self-efficacy, behavioral options, and their impact, including opportunities for action should be demonstrated [36]. Skills training should be organized to present orderly and clear instructions. Key messages should highlight relevance and usefulness, positive and negative examples [37], simple behaviors [38], pre-knowledge, transferability [39], and tailored information for the specific context [40]. Feedback is also needed to provide an indication of people’s effectiveness in making a difference [41].

### 4.4. Communication Guidelines to Promote Confirmation of Practicing Urban Agriculture

The EAST framework, developed by the Behavioural Insights Team [42], was applied in this part to analyze and synthesize how to promote confirmation of practicing urban agriculture. The framework consists of: (1) make it easy, (2) make it attractive, (3) make it social, and (4) make it timely.

(1) Make it easy: Practical agricultural knowledge should be provided by agricultural academic scholars, researchers, extensionists, and local experts in each community. The agricultural action knowledge should be simplified by using local language and a form of explanation based on the understanding of the local people, with clear, visualized, and tangible benefits, customized to each area of urban agriculture. Online and user-friendly urban agricultural knowledge management platforms for people to access at any time and place covering all important issues should be developed. Furthermore, peer-to-peer farming support and mentoring community partners located close to each other should be matched between the experienced and inexperienced communities, in order to share and learn with hands-on experience, including indigenous knowledge and resource sharing with mutual agreement on the resource allocation among the communities. 

(2) Make it attractive: Promote the benefits of urban agriculture, particularly to reduce expenditure on buying vegetables and fruits, and to earn more income for those who can achieve larger production and therefore have more products to sell. Moreover, food security for families should be highlighted covering food availability, food safety, food access, and food stability. These highlighted financial incentives can be an effective incentive for people to adopt and confirm the practice of urban agriculture.

(3) Make it social: To promote urban agriculture as a social issue, the Ministry of Agriculture and Cooperatives should launch policy and support seed grants to allocate area, materials, and facilities for practicing urban agriculture. In addition, emphasizing the type of urban agriculture that is appropriate for the area size and arrangement should be focused on so that people can see and decide based on their resources. The central and local media should also broadcast the success factors and stories, including lessons learned from the experienced communities to show how other people practiced and managed urban agriculture. Related agricultural organizations should cooperate to launch, campaign, and/or show a snapshot of practical knowledge on how to grow and support urban agriculture in any aspect. Practical and scientific knowledge should be shared between students in various fields from vocational schools and universities with urban agriculture villagers, as the technique of network power can enhance more learning and sharing in terms of agricultural management, marketing communication, knowledge, and technology transfer. Additionally, encouraging people to make a commitment to mentoring communities might be another technique to make this more socialized.

(4) Make it timely: People can be prompted by facilitating important action knowledge on urban agriculture to help the understanding of the required materials and facilities for each community, and the community members can share those inside their community, or even between the nearby communities. This can also help to reduce the costs of urban agriculture. Helping interested people to design and plan for starting urban agriculture, including the monitoring and assisting of those who already tried or adopted urban agriculture in order to solve their problems and sharing these experiences could assist and prompt people.

Critical Participatory Action Research (C-PAR) [43] should be applied as the process of communication design to promote the EAST framework as per the above details. C-PAR aims to facilitate understanding, practices, and conditions of practice. To understand people’s insight behavior, C-PAR highlights three elements driving behavior: (1) What is in people’s heads (the semantic space) relating to cultural–discursive arrangements, which can be observed through their sayings, and should be established based on the people’s understanding of urban agriculture practice? (2) What can be tangible and timely (the physical space and time) relating to material–economic arrangements, which can be observed through their doings, and what change agents/opinion leaders should be worked with initially to motivate other members to follow? Finally, (3) policy and regulations (the social space) relating to social–political arrangements, which can be observed through their relations, and contexts facilitating practice should be organized.

**Figure 4:**
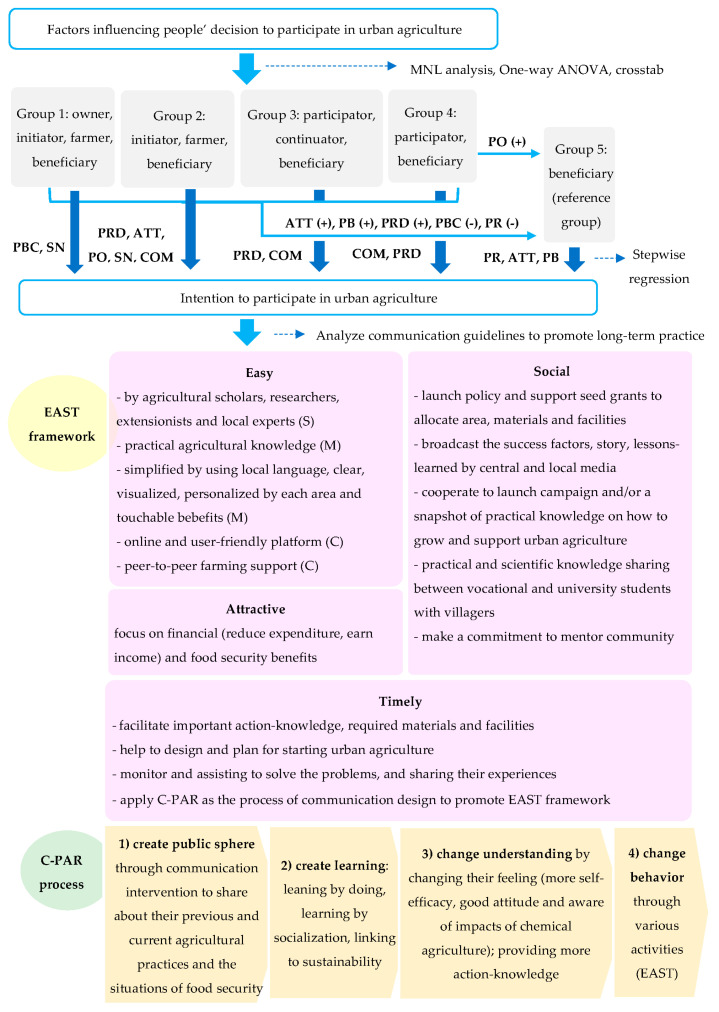
Communication guidelines to promote long-term practice of urban agriculture.

The C-PAR process can be designed to include four main steps. First, create the public sphere through communication intervention to share villagers’ previous and current agricultural practices and the situations of food security for themselves and their family members. The community members joining this intervention should be volunteers and feel free to participate, as well as respect each other. Second, create learning among community members and researchers by organizing the activities to promote: (1) leaning by doing—focusing on the action–knowledge and practical activities of how to start and do urban agriculture, (2) learning by socialization—focusing on onsite study visits, practical learning, and sharing ideas and experiences of urban agriculture, and (3) linking to sustainability: connecting with other partners to support urban agriculture. Third, change the understanding of community members by (1) changing their feeling so that they experience self-efficacy, a good attitude toward urban agriculture, and an awareness of the impacts of chemical urban agriculture; and (2) providing more action knowledge based on the needs of each community and their contexts. Lastly, change behavior through various activities linking to practicing urban agriculture based on the above EAST framework clarification. The conditions or benefits of promoting economic–environmental–social–health perspectives should be highlighted, such as reducing expenses from buying vegetables for cooking, gaining more income from selling vegetables, reducing food waste for a better environment, and strengthening human relationships, by sharing from their own planting products to others, and enjoying safe food for good health (Figure 4).

## 5. Conclusions

The results of MNL analysis emphasize that the factors of ATT, PB, and PRD were found to be a positive significant influence on urban agriculture adopting groups when the reference group was non-adopting respondents. Distance seemed to be an obvious factor influencing groups 1, 3, and 5. The nearest distance between group 1 and group 3 respondents’ households to their agricultural area presented as a key factor influencing their practice of urban agriculture, while the farthest distance could cause no participation for group 5 respondents. Key drivers influencing villagers’ intention to continue practicing urban agriculture for the initiator group owning their land were PBC and SN. PRD and COM were significant factors on the adoption of other groups. 

The EAST framework was applied to analyze and synthesize how to promote the confirmation of practicing urban agriculture. The communication guidelines that promote this continuation consists of four makes. First, make it easy by providing practical agricultural knowledge from agricultural scholars, extensionists, and local experts in each community with practice. The knowledge should be simplified by using local language, and visualized by infographics, customized for each area of interest, with illustrations of the tangible benefits. An online and user-friendly knowledge management platform that can be accessed at any time and place covering all important issues should be developed. Peer-to-peer farming support and mentoring community partners located close to each other should be matched between experienced and inexperienced communities in order to share and learn from hands-on experience and indigenous knowledge, including resource sharing with mutual agreement on the resource allocation among the communities. Second, make it attractive by emphasizing the obvious financial or economic benefits of urban agriculture, particularly that of the reduced expenditure on vegetables and fruits, and the earning of more income for those who can produce on a larger scale and have more products to sell. Comparison between adoption and non-adoption of urban agriculture can help promote visualization and realization. Third, make it social by implementing the policy and supporting seed grants to allocate area, materials, and facilities for practicing urban agriculture. The type of urban agriculture that is appropriate for the area size and arrangement should be focused on so that people can see and decide based on their resources. The central and local media should also broadcast success factors and stories, including lessons learned from the experienced communities, to show how other people practiced and managed urban agriculture. A campaign and/or a snapshot of practical urban agriculture knowledge on how to grow, take care of, and support urban agriculture in every aspect should be communicated. Practical and scientific knowledge sharing between students in various fields from vocational schools and universities as a demonstration of the power of networking can enhance more learning and sharing in terms of agricultural management, marketing communication, knowledge, and technology transfer. Moreover, making a commitment to mentoring communities might be another technique to make urban agriculture more socialized. Fourth, make it timely by facilitating important action knowledge on urban agriculture, materials, and facilities required for each community. Then, community members can share these inside their community or even between nearby communities, which can also help to reduce the costs of doing urban agriculture. Furthermore, helping interested people to design and plan for starting urban agriculture, as well as monitoring and assisting those who already tried or adopted urban agriculture to solve the problems and sharing their experiences, can prompt them into long-term practice. 

C-PAR should be applied as the process of communication design to promote the EAST framework. To understand people’s insight behavior, the C-PAR process can be applied covering four main steps. First, create a public sphere through communication intervention to share previous and current agricultural practices and the effects of food security. Second, create learning by organizing the activities to promote: (1) learning by doing, (2) learning by socialization, and (3) linking to sustainability. Third, change the understanding of community members by changing their feeling so that they experience greater self-efficacy, a positive attitude toward urban agriculture, and an awareness of the impacts of chemical urban agriculture, while providing more action knowledge based on the need of each community and their contexts. Lastly, change behavior through various activities linking to the practice of urban agriculture based on the above EAST framework clarification. The conditions and benefits of promoting economic–environmental–social–health perspectives should be highlighted.

A limitation of this study was that actually there are many groups of people practicing urban agriculture. This study was trying to analyze from the groups and communities with continuous practice and put their attempts to deal with difficulties and challenges during their urban agriculture projects as recommended by the initiators of urban agriculture in Thai society. 

Future research should highlight monitoring, mentoring, and empowering the urban agriculture groups and communities to ensure their continuous practice in the long run. If they face some problems, the monitoring, mentoring, and empowering process by the urban agriculture experts from academic institutions and agricultural organizations should be provided. The user-friendly and up-to-date online knowledge platform should be developed to be more conveniently accessible by the interested people at any time. The two-way communication should be employed so that the people can input their questions together with photos, if available, and the quick response should be considered as well. 

## Figures and Tables

**Table 1:** Psychological factors affecting urban agriculture, reliability test, and reference theories.

Psychological Factors towards Urban Agriculture	Cronbach’s Alpha	Reference Theories	Sources
(1) Attitude (ATT)	0.858	TPB, TRA	[7,8]
(2) Social Norm (SN)	0.964	TPB	[7,8]
(3) Perceived Behavioral Control (PBC)	0.958	TPB	[6,20]
(4) Perceived Benefits (PB)	0.928	HBM, TRA	[19,20]
(5) Perceived Obstacles (PO)	0.890	HBM	[7]
(6) Perceived Risks (PR)	0.943	HBM	[19] (based on the study areas)
(7) Cue to action: Communication (COM)	0.925	HBM	based on the study areas
(8) Self-efficacy: Perceived Readiness (PRD)	0.961	HBM	[7] (based on the study areas)
(9) Intention (ITT)	0.845	TPB, TRA	[6,20]

**Table 2:** The questions in the questionnaire.

Part	Questions
(1) Demographic information (a check list and an open form)	1.1 Gender (1. male, 2. female) 1.2 Age (indicating years) 1.3 Schooling (indicating years) 1.4 Farmland owner (1. no, 2. yes) 1.5 Start date of urban agriculture (indicating month and year) 1.6 Reasons for practicing urban agriculture (explaining the reasons)
(2) Social and physical distance factors (a check list and an open form)	2.1 Accommodation characteristics (1. detached house, 2. townhomes/townhouses, 3. rented rooms, 4. schools/temples) 2.2 Distance to the farming area (1. <100 m, 2. 100–500 m, 3. >500–2000 m, 4. >2000 m) 2.3 Characteristics of cultivated agricultural areas (1. next to/in front of/back of the house, 2. agricultural area in the office/school/temple, 3. rooftop, 4. terrace, 5. community acquaintance area, 6. community public area) 2.4 Participation in urban agriculture (1. farmland owner–initiator–farmer–beneficiary, 2. initiator–farmer–beneficiary, 3. Participator–continuator–beneficiary, 4. participator–beneficiary, 5. beneficiary)
(3) Psychological factors (a five-point Likert scale answer: 5 = most, 4 = more, 3 = moderate, 2 = low, 1 = very low)	3.1 Attitude (ATT) ATT1: Urban agriculture helps improve the environment. ATT2: Urban agriculture helps reduce food costs. ATT3: Urban agriculture helps contribute to food security. ATT4: Urban agriculture helps strengthen relationships in the community. ATT5: Urban agriculture is linked to the use of water–energy–food–people (WEFP) resources.
	3.2 Social norm (SN) SN1: People close to me want me to undertake urban agriculture. SN2: People close to me agree with me to continue practicing urban agriculture.
	3.3 Perceived behavioral control (PBC) PBC1: I am sure I can undertake urban agriculture. PBC2: I have personal control of urban agriculture. PBC3: I undertake urban agriculture, or not—it is totally up to me.
	3.4 Perceived benefits (PB) PB1: Urban agriculture means that I have eaten safe food. PB2: Urban agriculture meants that I take exercise and improve my physical health. PB3: Urban agriculture means that I use my free time more constructively. PB4: Urban agriculture helps reduce food waste. PB5: Urban agriculture helps relieve stress. PB6: Urban agriculture helps generate income. PB7: Urban agriculture provides the opportunity to learn more. PB8: Urban agriculture helps boosts self-confidence. PB9: Urban agriculture helps creates added value.
	3.5 Perceived obstacles (PO) PO1: Not having enough time for urban agriculture. PO2: Lacks the convenience of traveling to the agricultural area. PO3: I need to take care of my family (family responsibility). PO4: Lack of knowledge. PO5: Not taking it seriously. PO6: Lack of resources (e.g., farming area, water, people, seeds).
	3.6 Perceived risks (PR) PR1: Crops from urban agriculture attract insects that may be harmful to humans. PR2: Urban agriculture causes traffic jams because of agricultural machines and waste on the road. PR3: Urban agriculture is detrimental to the natural environment, resulting in soil and water contamination. PR4: Urban agriculture hinders the expansion of urban areas causing economic development to slow down. PR5: Urban agriculture poses a threat to human health. PR6: Urban agriculture spoils the urban landscape.
	3.7 Communication (COM) COM1: Trusted agricultural experts/experienced farmers received on-site practical training and study visits. COM 2: Trusted agricultural experts/experienced farmers received virtual practical training and study visits. COM3: Proper and visualized urban agriculture techniques were demonstrated in on-site practical training and study visits. COM4: Proper and visualized urban agriculture techniques received virtual practical training and study visits. COM5: On-site practical training and study visits were accessible. COM6: Virtual practical training and study visits were accessible. COM7: Your satisfaction with, and convince by, the on-site practical training and study visits from the agricultural experts/experienced farmers. COM8: Your satisfaction with, and convince by, the information from the agricultural experts/experienced farmers during the practical training and study visits.
	3.8 Perceived readiness (PRD) PRD1: You are ready for urban agriculture in terms of the area. PRD2: You are ready for bio-fertilizer to nourish the soil. PRD3: You are ready for water resources in urban agriculture. PRD4: You are ready to use alternative energy in urban agriculture. PRD5: You are ready for human resources in urban agriculture. PRD6: You have seed availability in urban agriculture. PRD7: You are ready in terms of materials and equipment for farming in the city. PRD8: You are ready in terms of your knowledge of urban agriculture.
	3.9 Intention (ITT) ITT1: I intend to undertake urban agriculture without chemicals. ITT2: I intend to nourish the soil using natural compost. ITT3: I intend to seek more knowledge in order to be successful in urban agriculture. ITT4: If obstacles are encountered, I will find a solution to those obstacles and continue farming in the city.

**Table 3:** Classification.

Observed	Predicted					
	1 Owner, Initiator, Farmer, Beneficiary	2 Initiator, Farmer, Beneficiary	3 Participator, Continuator, Beneficiary	4 Participator, Beneficiary	5 Beneficiary	Percent Correct
1 owner, initiator, farmer, beneficiary	22	4	2	0	0	78.6%
2 initiator, farmer, beneficiary	4	42	7	0	0	79.2%
3 participator, continuator, beneficiary	0	2	46	5	2	83.6%
4 participator, beneficiary	0	0	4	30	0	88.2%
5 beneficiary	0	0	2	0	153	98.7%
Overall Percentage	8.0%	14.8%	18.8%	10.8%	47.7%	90.2%

**Table 4:** The likelihood ratio tests.

Effect	Model Fitting Criteria	Likelihood Ratio Tests		
	−2 Log Likelihood of Reduced Model	Chi-Square	df	Sig.
Intercept	238.576	62.466	4	0.000
ATT	214.302	38.193	4	0.000
SN	202.069	25.960	4	0.000
PBC	257.306	81.196	4	0.000
PB	229.694	53.584	4	0.000
PR	246.629	70.520	4	0.000
PO	249.753	73.644	4	0.000
PRD	259.229	83.119	4	0.000
COM	196.597	20.487	4	0.000

The chi-square statistic is the difference in −2 log-likelihoods between the final model and a reduced model. The reduced model is formed by omitting an effect from the final model. The null hypothesis is that all parameters of that effect are 0.

**Table 5:** The marginal effect results of factors influencing people’s decision to participate in urban agriculture (the reference category is group 5 respondents).

Dependent Variables	Model 1	Model 2	Model 3	Model 4
	Exp(B)	Exp(B)	Exp(B)	Exp(B)
ATT	6,368,265.704 ***	853,810.774 **	2,396,974.203 **	1,077,220.039 **
SN	0.037	0.854	0.976	3.135
PBC	2.962 × 10^−7^ **	8.528 × 10^−8^ **	7.339 × 10^−9^ **	2.604 × 10^−6^ *
PB	55,628,264.015 **	352,811,535.239 **	1,352,429.342 *	233,157.646 *
PR	5.314 × 10^−9^ *	4.566 × 10^−9^ *	7.699 × 10^−11^ **	7.554 × 10^−10^ *
PO	8.257	7.519	0.040	44.602 *
PRD	638,201,298.217 ***	68,255,949.265 **	1,076,712.577 **	173,003.759 *
COM	3.363	1.300	32.800	4.311

Note: *** *p* value < 0.001, ** *p* value < 0.01, and * *p* value < 0.05. The green box means a positive value, and the orange box means a negative value.

**Table 6:** Classification of decision tree analysis.

Observed	Predicted					
	1 Owner, Initiator, Farmer, Beneficiary	2 Initiator, Farmer, Beneficiary	3 Participator, Continuator, Beneficiary	4 Participator, Beneficiary	5 Beneficiary	Percent Correct
1 owner, initiator, farmer, beneficiary	26	0	0	0	2	92.86%
2 initiator, farmer, beneficiary	0	0	22	0	31	0.00%
3 participator, continuator, beneficiary	4	0	24	0	27	43.64%
4 participator, beneficiary	17	0	0	0	17	0.00%
5 beneficiary	16	0	8	0	131	84.52%
Overall Percentage	19.4%	0.0%	16.6%	0.0%	64.0%	55.69%

Growing Method: CHAID, dependent variable: participation.

**Table 7:** Risk.

Estimate	Std. Error
0.4431	0.0276

Growing Method: CHAID, dependent variable: participation.

**Table 8:** Stepwise multiple linear regression.

Group				Collinearity Statistics	
		t	Sig.	Tolerance	VIF
1	(Constant)				
	PBC	7.669	0.000	0.224	4.455
	SN	2.256	0.033	0.224	4.455
2	(Constant)	0.904	0.371		
	PRD	3.310	0.002	0.611	1.637
	ATT	2.587	0.013	0.505	1.980
	PO	−6.665	0.000	0.494	2.024
	SN	3.218	0.002	0.507	1.974
	COM	2.170	0.035	0.725	1.379
3	(Constant)	7.993	0.000		
	PRD	5.906	0.000	0.574	1.741
	COM	2.225	0.030	0.574	1.741
4	(Constant)	−0.144	0.887		
	COM	3.609	0.001	0.954	1.049
	PRD	2.907	0.007	0.954	1.049
5	(Constant)	−3.391	0.001		
	PR	4.653	0.000	0.665	1.505
	ATT	5.690	0.000	0.699	1.431
	PB	5.383	0.000	0.942	1.061

## Data Availability

Not applicable.
